# Chromosomal Microarray Analysis versus Karyotyping in Fetuses with Increased Nuchal Translucency

**DOI:** 10.3390/medsci7030040

**Published:** 2019-02-27

**Authors:** Rita Cicatiello, Piero Pignataro, Antonella Izzo, Nunzia Mollo, Lucia Pezone, Giuseppe Maria Maruotti, Laura Sarno, Gabriella Sglavo, Anna Conti, Rita Genesio, Lucio Nitsch

**Affiliations:** 1 Dept. Molecular Medicine and Medical Biotechnology, School of Medicine, University of Naples Federico II, 80131 Naples, Italy; rita.cicatiello@unina.it (R.C.); piero.pignataro@gmail.com (P.P.); antonella.izzo@unina.it (A.I.); nunzia.mollo@unina.it (N.M.); pezonel87@gmail.com (L.P.); rgenesio@unina.it (R.G.); nitsch@unina.it (L.N.); 2 Maternal-Child Department, School of Medicine, University of Naples Federico II, 80131 Naples, Italy; gm.mar@tiscali.it (G.M.M.); laura.sarno@unina.it (L.S.); gabriella.sglavo@unina.it (G.S.); 3 Institute of Experimental Endocrinology and Oncology, National Research Council, 80131 Naples, Italy

**Keywords:** nuchal translucency, chromosome microarray analysis, non-invasive prenatal testing, prenatal diagnosis

## Abstract

We have carried out a retrospective study of chromosome anomalies associated with increased nuchal translucency (NT) in order to compare yield rates of karyotype, chromosome microarray analysis (CMA), and non-invasive prenatal testing (NIPT) in this condition. Presenting with increased NT or cystic hygroma ≥3.5 mm as an isolated sign, 249 fetuses underwent karyotype and/or CMA from 11 to 18 gestational weeks. Karyotype and fluorescence in situ hybridization (FISH) analyses detected 103 chromosomal anomalies including 95 aneuploidies and eight chromosomal rearrangements or derivatives. Further, seven pathogenic copy number variants (CNV), five likely pathogenic CNVs, and 15 variants of unknown significance (VOUS) were detected by CMA in fetuses with normal karyotype. Genetic testing is now facing new challenges due to results with uncertain clinical impacts. Additional investigations will be necessary to interpret these findings. More than 15% of the anomalies that we have diagnosed with invasive techniques could not be detected by NIPT. It is therefore definitely not recommended in the case of ultrasound anomalies. These results, while corroborating the use of CMA in fetuses with increased NT as a second tier after rapid aneuploidy testing, do not suggest a dismissal of karyotype analysis.

## 1. Introduction

Nuchal translucency (NT) analysis is an ultra-sonographic prenatal scan carried out at 11 − 13 + 6 weeks of gestation to assess the quantity of fluid present within the nape of the fetal neck. It is useful to predict chromosomal abnormalities as well as cardiovascular abnormalities in a fetus [1]. Nuchal translucency measurements were initially used as a stand-alone test for aneuploidy screening. Maternal age was then added, and finally, NT became part of a combined first trimester aneuploidy screening tool (NT, maternal age, and up to five maternal serum markers) [2]. Nuchal translucency above the 99th percentile (≥3.5 mm) is associated with a high risk for more than 50 genetic conditions such as chromosomal abnormalities, fetal malformations, major cardiac defects, certain genetic syndromes (diGeorge syndrome, Noonan syndrome, and Smith-Lemli-Opitz syndrome), and fetal or perinatal death [3,4,5]. An increased NT value is therefore an indication for invasive prenatal cytogenetic analysis. 

Several studies have demonstrated the advantages of implementing chromosomal microarray analysis (CMA) in the prenatal setting for the evaluation of fetuses with major structural anomalies [6]. The evolution from karyotype and fluorescence in situ hybridization (FISH) to CMA allowed for the identification of deletions and duplications previously unrecognized, making available the diagnosis of an increased spectrum of cytogenetic anomalies. A recent meta-analysis performed in fetuses with isolated, increased NT showed that CMA can detect about 4% of pathogenic copy number variants (CNVs) not visible by karyotyping [6].

The introduction of non-invasive prenatal testing (NIPT), based on the analysis of cell-free fetal DNA in maternal blood [7], proved to be very efficient in pregnant women at high risk of aneuploidy. Further validation studies were required in populations at low-risk [8]. The best prerogatives of NIPT are excellent sensitivity and specificity for common autosomal aneuploidies (13, 18, and 21 trisomies), and to a lesser extent, for sex chromosome aneuploidies [9] at a very early gestational age. The purpose of the present study was to analyze chromosome anomalies associated with increased NT in order to compare yield rates of karyotype and CMA and to speculate about NIPT efficiency in this condition. To achieve this aim, a retrospective study was carried out considering 595 fetuses that underwent karyotype and/or CMA in our cytogenetics unit after detection of ultrasound anomalies in the last three years. 

Fetuses, 249 in number, with increased NT or cystic hygroma as an isolated sign were selected for this study. We discuss the results of this study and have the opportunity to use one technique or another in the case of increased NT above the 99th percentile. Considerations about NIPT generally refer to virtual simulations of what is detectable by this technique. 

## 2. Materials and Methods

Fetuses with a nuchal translucency or cystic hygroma ≥3.5 mm, as isolated signs, underwent karyotype and/or CMA from 11 to 18 gestational weeks. Depending on the gestational week, cytogenetic analysis was performed on samples from 169 chorionic villi (CVS) and 80 amniotic fluid (AF). Maternal age ranged from 18 to 43 years.

Rapid FISH analyses were performed as first tier using XA Aneuscore II Probe Kit (MetaSystems, Milano, Italy) according to the manufacturer’s standard protocols to recognize the most common aneuploidies (13, 18, 21, X, and Y chromosomes).

When the FISH result was normal or when a genomic characterization was needed, CMA was performed as a second tier. DNA samples were hybridized on PerkinElmer or Agilent HD 4 × 180 K chips (PerkinElmer, Wallac, Turku, Finland; Agilent technologies Italia SpA, Cernusco sul Naviglio, MI Italy) in accordance with the manufacturer’s guidelines for the whole genome screening. The arrays were scanned by the Agilent SureScan Dx Microarray Scanner and analyzed using Cytogenomics (Agilent technologies Italia SpA, Cernusco Sul Naviglio—MI) and Genoglyphix softwares (Signature Genomics, Spokane, WA, USA), referring to the GRCh37/Hg19 Genome Assembly. To confirm the pathogenic CNVs FISH analysis on metaphases from the fetus and from the parents (to determine their inheritance) was performed using locus specific probes from Bacterial Artificial Chromosome libraries (Empire genomics, Williamsville, NY, USA) or SureFISH probes chemically synthesized using oligonucleotide library synthesis technology (Agilent technologies Italia SpA).

Karyotype analysis was performed on GTG-banded metaphases from AF (in situ clones obtained after 8–10 days of culture) or CVS (short and long-term cultures) at a resolution of 400 bands according to standard cytogenetic protocols. 

Chromosome rearrangements and markers were characterized using whole chromosome painting or locus specific probes (Kreatech, Milano, Italy). Multicolor FISH (M-FISH), using the 24XCyte (Human Multicolor FISH Probes, Metasystems) kit, and multicolor banding (MCB), using the multicolor banding DNA Probe Kit (MetaSystems) were also performed when needed. All FISH analyses were performed according to the manufacturer’s protocols. Chromosome images were analyzed using the Axio Imager Z1 mot microscope (Carl Zeiss SpA, Milan, Italy) with the IKAROS and ISIS software imaging system (Metasystems, Altlussheim, Germany).

Results of CMA were retrospectively reanalyzed and CNV size, genomic position, as well as gene content were evaluated for clinical significance. The published literature and public databases were also consulted to facilitate the interpretation of detected CNVs. These were classified as pathogenic when: (a) Pathogenicity, associated with the region, was assessed in public databases and literature (Online Mendelian Inheritance in Man (OMIM) database—http://www.ncbi.nlm.nih.gov/omim; Database of Chromosomal Imbalance and Phenotype in Humans using Ensembl Resources (DECIPHER)—https://decipher.sanger.ac.uk/; ClinVar—https://www.ncbi.nlm.nih.gov/clinvar/); and (b) there was evidence in ClinGen or literature that a dosage-sensitivity may result in a clinically abnormal phenotype (ClinGen Dosage Sensitivity Map—https://www.ncbi.nlm.nih.gov/projects/dbvar/clingen). CNVs were classified as likely pathogenic according to literature reports. 

Finally, CNVs were considered variants of unknown significance (VOUS) when: (a) The chromosomal region contained no gene or transcript at all; (b) there was no report of a clinically abnormal phenotype associated to them; (c) dosage sensitivity for genes mapping to the region was unlikely or not demonstrated; and (d) CNV did not meet the criteria for classification as pathogenic or benign [10].

Karyotype, FISH, and CMA reports in this paper are made according to the International System of Human Cytogenetic Nomenclature (ISCN) 2016. Informed consent for genetic studies was obtained from all pregnant women during a pre-test genetic counseling session in which we also collected information about anamnestic and clinically relevant data. During counseling, women chose which type of CNV should be included in the final report.

Noninvasive test was not performed in most of the cases, and when performed, it was not carried out in our laboratory. Detection rates were calculated according to theoretical assumptions of what is detectable with high predictive value by the current NIPT technologies. 

## 3. Results

This was a retrospective study of 595 fetuses that underwent invasive prenatal diagnosis after detection of ultrasound anomalies between January 2015 and July 2018 at the Federico II Medical School, University of Naples, Italy. We selected 249 of them with NT or cystic hygroma above the 99th percentile in order to analyze chromosome anomalies associated with increased NT and to evaluate the detection rate of different diagnostic techniques.

Overall, 110 (~44%) chromosomal abnormalities were detected by cytogenetic and cytogenomic analyses. In detail, karyotype and FISH analyses detected 103 chromosomal anomalies including 95 aneuploidies involving chromosomes 9, 13, 18, 21, X and 8 chromosomal rearrangements or derivatives. Further 7 pathogenic copy number variants (CNV) were detected by CMA in fetuses with normal karyotype. (Table 1)

The chromosome rearrangements included three unbalanced translocations, one inversion, and four markers visible to karyotype, which needed further investigation by different techniques (FISH and CMA). Only 5 of them were correctly characterized by CMA. Three rearrangements were undetectable by CMA: One of them appeared balanced at the CMA resolution we used and two included regions not represented on the chip array. These rearrangements were characterized by multicolor and multi-banding FISH.

High-resolution CMA was performed on a total of 155 cases with normal rapid FISH at a mean between 25 and 75 Kb resolution in targeted regions and 150 Kb in the backbone. The size of CNV, its genomic position, as well as gene content were reconsidered for clinical significance according to recent published literature and public databases.

Overall, CMA unraveled 13 pathogenic CNVs (eight of which were not visible and five were visible to karyotype), five likely pathogenic ones, and 15 inherited VOUS. Furthermore, CMA was useful to better clarify the approximate breakpoint positions in the case of unbalanced rearrangements and unraveled a pathogenic 6 Mb deletion in the p arm of chromosome eight in which karyotype analysis evidenced only a duplication (Table S1). 

Cryptic pathogenic CNVs detected in this study were associated with the following clinically relevant anomalies: Two 22q11.2 deletions associated with DiGeorge syndrome (MIM #188400); two 16p11.2 duplications associated with proximal or distal 16p11.2 micro-duplication syndrome (MIM #614671); 1p36 deletion syndrome (MIM #607872); 1q21.1 duplication syndrome (MIM #612475); and RYR2 deletion associated to heart anomalies (MIM #600996, MIM #604772). 

Finally, chromosomal abnormalities were examined in order to evaluate their detectability in the case of NIPT analysis.

We presumed that cryptic chromosomal anomalies, aneuploidies of chromosomes that are different from 13, 18, 21, X, and Y, together with marker chromosomes and unbalanced rearrangements that we have identified by karyotype and/or CMA, could not be detected by NIPT for a total amount of 16/110 (~15%) chromosomal abnormalities. In addition, three 45 X mosaicisms at very low percentages were likely not detectable by the technique (Table 2), while 11 homogeneous X monosomies and one mosaic X monosomy at high percentages were likely to be detectable with a predictive value lower than the other aneuploidies [9].

## 4. Discussion

In the last years, several studies have demonstrated the advantages of implementing microarray technologies in the prenatal setting. Many studies have investigated the possible association between increased NT and submicroscopic chromosomal abnormalities detected by CMA [6,11] and appropriate guidelines have been issued. The amount of pathogenic sub-microscopic CNVs in fetuses with ultrasound anomalies reported in the literature is around 6–8% of cases, especially when cardiac, skeletal, urogenital, and central nervous system abnormalities are associated [6]. However, how and when to use this technique in clinical practice and what resolution must be applied is still a matter of discussion. Some authors propose CMA analysis after karyotyping when NT is ≥3.5 mm and/or ultrasound examination shows other fetal abnormalities. The American College of Obstetricians and Gynecologists (ACOG) suggest that the usefulness of microarray as the first-line test for prenatal evaluation of chromosomal abnormalities remains unknown and conventional karyotyping remains the primary cytogenetic tool [12]. Other authors suggest that CMA should replace karyotyping in prenatal testing where invasive procedures are required [11,13].

In our study, 94 aneuploidies in 249 fetuses with increased NT were detectable in 48 h using FISH on interphase nuclei. Therefore, for economic reasons and rapidity of the report, we suggest performing at least a rapid diagnostic test for aneuploidies before proceeding to the CMA. The remaining 155 prenatal samples were investigated by CMA with an increase in the detection rate of 5% if we consider only the pathogenic CNVs. It rose up to 8% if we also consider the likely pathogenic ones. 

Worthy of attention in the prenatal setting are CNVs predisposed to neuro-developmental disorders (i.e., CNVs with incomplete penetrance and variable expressivity) [6]. Variants in the 16p11.2 region can be associated with neuro-developmental disorders including autism spectrum disorders, schizophrenia, intellectual disability, microcephaly, facial dysmorphism [6,14], and obesity [15], even though 16p11.2 duplications or deletions can also be found in asymptomatic carriers [16]. Since the neuro-developmental phenotype cannot be determined in the prenatal setting, these CNVs raise significant challenges for genetic counseling. For this reason, some laboratories do not report them in the prenatal setting and the corresponding CMA probes are not included in some targeted prenatal arrays. We decided to report the CNVs as pathogenic, highlighting the incomplete penetrance and variable phenotype in both the written report and during the post-test genetic counseling. 

In this study, five likely pathogenic CNVs and 15 VOUS were detected. According to a specific woman’s consent to having CNV be reported, we did not communicate these results except for a de novo deletion in the chromosomal band 9p24.3 overlapping the OMIM gene *DOCK8*. Recent reports describe, in carriers of *DOCK8* deletions, variable clinical manifestations such as intellectual disability, developmental delay, facial dysmorphic features, autism spectrum disorders, and psychiatric behavior [17]. Additionally, duplications encompassing the *DOCK8* gene are associated with anomalies in psychiatric behavior [18]. We reported this de novo CNV as likely pathogenic on the basis of literature. The other likely pathogenic CNVs only partially overlap genes that were reported to be associated with pathologies, but the clinical impact of deletions or duplications of parts of these genes are unknown.

Karyotype analysis and/or FISH allowed for the obtaining of a cytogenetic diagnosis not detectable by CMA in six cases, including one that apparently balanced de novo inversion, two supernumerary marker chromosomes derived from chromosome nine, and three X monosomies with low-level mosaicism. 

Even though the detected rearrangement and the marker chromosomes were considered to be likely non-pathogenic in the genetic counseling, it cannot be completely excluded that they might have some relevance. Balanced rearrangements apparently may cause phenotypic alterations due to position effects [19] or disruption of regulatory elements such as enhancers, promoters, locus control regions, and topological associated domains (TAD) boundaries, which can lead to disease-related changes in gene expression [20]. Especially when the rearrangement is de novo and a phenotypic anomaly is detected (like the case we report), information about these possibilities, although rare, must be included in the post-test genetic counseling. Furthermore, if the rearrangement falls very close to a fundamental gene, a sequencing of the region might give useful information.

Marker chromosomes, undetectable by CMA, involved ethero-chromatic regions of chromosome nine. The prognosis in these cases was favorable, especially for one maternal inherited marker. However, a marker, even an undetectable one, may mask a rescued trisomy. Therefore, in the case of markers involving imprinted chromosomes, like 7, 11, 14, or 15, it should be advisable to extend the analysis to exclude uni-parental disomy with pathogenic consequences.

Mosaic aneuploidies reported in this study involved the X chromosome and they could be, of course, detected by rapid FISH, but this is not true in the case of low-level mosaicisms of chromosomes different from 13, 18, 21, X, or Y that may cause consequences even worse than mosaic X monosomy. In this case, only the karyotype makes the difference.

Overall, these events indicate that there are chromosome anomalies not detected by either CMA or by rapid FISH. It is likely that some of them may have a higher pathogenic relevance with respect to the ones we have found, which also require a diagnostic follow-up. For these reasons, we do not agree with the proposal that CMA can completely replace karyotype analysis.

Genetic testing now faces new challenges due to results with uncertain clinical impact. All these reports highlight the importance of better defining the penetrance of genetic variants. The above argumentation about TAD may be applied to uncertain or likely pathogenic CNVs. Unfortunately, TAD analysis might not be appropriate as a diagnostic tool in prenatal diagnosis since genome organization structure may differ in different cell types and different developmental stages [20]. Amniocytes are representative of a heterogeneous cell population and the chorion villi developmental stage might not be informative. It may therefore be difficult to draw conclusions regarding the possible phenotype of an unborn child starting from such experimental data.

For what concerns the NIPT, it is definitely not recommended in the case of ultrasound anomalies, including isolated, increased NT above the 99th percentile, as, in our study, it would be unable to detect at least 15% of the anomalies diagnosed by the other techniques. Other authors demonstrated that, if NIPT is used as an alternative to invasive testing, there is a substantial risk that a pathogenic CNV will be missed or only identified later in pregnancy [13]. Literature highlights the critical need for comprehensive pre-test and post-test counseling as many patients may not recognize the potential for mosaicism, maternal or fetal, to be identified, or that other aneuploidies may be the true underlying diagnosis [9].

We believe that it is important that this information is included in pre-test counseling, especially if the risk of identifying a pathogenic CNV is higher than the simple risk of trisomy 21. 

## 5. Conclusions

In conclusion, in our experience, CMA demonstrated 5–8% incremental yield of CNV detection in fetuses with increased NT without other ultrasound anomalies and normal karyotype. For CMA, we suggest using a high resolution, not targeting, arrays (at least 180 K) to preserve the possibility to exclude uncertain CNVs during the interpretation of results, which also takes into account the observed phenotype and family history. On the other hand, the karyotype demonstrated its importance because identified markers and structural anomalies were not detected by CMA. We therefore suggest, in these cases, to perform rapid FISH as the first tier to exclude common aneuploidies and then CMA together with karyotype analysis. This study also suggests that NIPT only represents an acceptable alternative to invasive diagnostic testing in the absence of any ultrasound anomaly including increased NT.

## Figures and Tables

**Table 1:** Chromosomal abnormalities detected by cytogenetic analysis and chromosomal microarray analysis (CMA).

Chromosomal Abnormalities	Total Cases
Trisomy 21	57
Trisomy 18	18
Trisomy 13	4
Trisomy 9	1
45,X	11
Mos 45,X	4
Chromosomal markers	4
Unbalanced rearrangements	3
Likely balanced rearrangement	1
CMA with submicroscopic pathogenic CNVs	7
Total	110

CNV: copy number variants.

**Table 2:** Prenatal cases with chromosome anomalies not detectable by non-invasive prenatal testing (NIPT).

Chromosome Anomaly	Sample Type	Detectable by NIPT
46,XX.arr[GRCh37]16p11.2(29652999_30198600)x3 pat	AF	No
46,XY.arr[GRCh37]2p15(61529343_61564040)x1 mat,16p11.2(28833437_29046252)x3 dn	AF	No
46,XX.arr[GRCh37]22q11.2(19010936_20434800)x1 dn	CVS	No
46,XX.arr[GRCh37]1p36.23p36.33(453255_7284969)x1 dn	CVS	No
46,XX.arr[GRCh37]22q11.2(19110226_19854855)x1 dn	CVS	No
46,XY.arr[GRCh37]1q43(237335376_237418407)x1 dn	AF	No
46,XY.arr[GRCh37]1q21.1(145429097_146756493)x3 pat	AF	No
47,XX,+9	AF	No
47,XY,+mar .ish der(9)(wcp9+)mat.arr[GRCh37](1-22)x2,(X,Y)x1	CVS	No
47,XY,+mar.ish der(22)(wcp22+,TUPLE1+).arr[GRCh37]22q11.1q11.21(17374086_20088001)x3 dn	CVS	No
47,XY,+mar .ish der(9)(wcp9+).arr[GRCh37](1-22)x2,(X,Y)x1	CVS	No
mos 47,XX,+mar[8]/46,XX[8].ish i(12)(p10)(wcp12+)	CVS	No
mos 46,XX,inv(5)(p14p15)[7]/46,XX[9].arr[GRCh37]8q22.2(100072320_100155410)3 pat	CVS	No
45,XY,der(18;22)(p11?;q11?).ish der(18;22)(D18Z1+,D18S552;TUPLE1+,SHANK3+)dn.arr[GRCh37]6q16.1(95588523_95662060)x1 pat,18p11.32p11.21(146484_14117327)x1	AF	No
46,XX,add(8)(p23).ish dup(8)(p21?p23)(wcp8+)dn.arr[GRCh37]8p23.3p23.1(228758_6911631)x1,8p23.1p11.22(11858401_38964086)x3	AF	No
46,XX,der(10)t(10;12)(q26;q23).arr[GRCh37]10q26.13q26.3(123190101_135104747)x1,12q23.3q24.33 (106838149_132878426)x3	CVS	No
Short term culture: 46,XY/Long term culture: mos 45,X[4]/46,XY[12]	CVS	Not sure
mos 45,X[4]/46,XX[26]	CVS	Not sure
mos 45,X[3]/46,XX[22]	CVS	Not sure

AF: amniotic fluid; CVS: samples from chorionic villi.

## Supplementary Materials

The following are available online at https://www.mdpi.com/2076-3271/7/3/40/s1. Table S1: Copy number variants detected in fetuses with isolated NT ≥ 3.5 mm.
