# Supplementary material for: Chromosomal Microarray Analysis versus Karyotyping in Fetuses with Increased Nuchal Translucency
Source: Med Sci (Basel). 2019 Feb 27;7(3):40. doi: 10.3390/medsci7030040 (PMC6473420; doi:10.3390/medsci7030040)
Supplement: Supplementary file 1 [file medsci-07-00040-s001.pdf]

Table S1. Copy number variants detected in fetuses with isolated NT ≥3.5 mm.

ID	Pathogenic CNVs	Description and size of CNVs
8175	18p11.32p11.21(146,484_14,117,327)x1	13.97 Mb Deletion due to an unbalanced de novo translocation containing 44 OMIM genes.
8891	8p23.3p23.1(228758_6911631)x1	6.68 Mb Deletion, due to de novo 8p rearrangement, containing 16 OMIM genes.
8891	8p23.1p11.22(11858401_38964086)x3	27 Mb Duplication, due to de novo 8p rearrangement, containing 135 OMIM genes.
9021	16p11.2(29652999_30198600)x3 pat	545 Kb Duplication, paternal origin, overlapping the critical region for 16p11.2 proximal microdup syndrome (BP4-BP5)
8521	16p11.2(28833437_29046252)x3 dn	de novo 217 Kb Duplication overlapping the critical region for 16p11.2 distal microdel. syndrome (BP2-BP3).
7733	22q11.2(19010936_20434800)x1 dn	de novo 1.4 Mb Deletion overlapping the critical region for 22q 11.2 microdeletion syndrome.
8442	22q11.2(19110226_19854855)x1 dn	de novo 744 Kb Deletion which partially overlaps the critical region for 22q 11.2 microdeletion syndrome.
7433	1p36.23p36.33(453255_7284,969)x1 dn	de novo 6.8 Mb Deletion overlapping the critical region for 1p36 microdeletion syndrome.
8009	10q26.13q26.3(123190101_135104747)x1	12 Mb Deletion, due to maternal balanced translocation, overlapping the critical region for 10q26 microdel. syndrome.
8009	12q23.3q24.33(106838149_132878426)x3	26 Mb Duplication, due to maternal balanced translocation, containing *PTPN11*, *TBX3* and *TBX5* genes.
8823	1q43(237335376_237418407)x1 dn	de novo 83 Kb Deletion of the 1q43 region containing the OMIM gene *RYR2*.
8035	1q21.1(145429097_146756493)x3 pat	1.3 Mb Duplication, paternal origin, which partially overlaps the critical region for 1q21.1 microdeletion syndrome.
	**Likely pathogenic CNVs**	
8968	9p24.3(222330_434797)x3 dn	de novo 212 Kb Duplication containing the OMIM gene *DOCK8*.
8875	10q21.3(68359435_68415649)x1 pat	56 Kb Deletion, paternal origin, containing the OMIM gene *CTNNA3*.
9001	Xq28(154124170_154233022)x2 dn	de novo 109 Kb Duplication of the Xq28 region overlapping part of the OMIM gene *F8*.
8931	3p22.1(41695444_41827282)x1 mat	132 Kb Deletion, maternal origin, containing the gene *ULK4*.
8931	20p12.1(15079518_15138764)x1 mat	59 Kb Deletion, maternal origin, containing the *MACROD2* gene.
	**Uncertain CNVs**	
8175	6q16.1(95588523_95662060)x1 pat	73 Kb Deletion, paternal origin.
8968	11p14.3(24522033_24617004)x3 mat	94 Kb Duplication, maternal origin.
8521	2p15(61529343_61564040)x1 mat	34 Kb Deletion, maternal origin, containing one OMIM gene.
8035	7q21.11(84709626_84813120)x3 pat	103 Kb Duplication, paternal origin, containing one OMIM gene.
8035	12p13.31(7986555_8113851)x3 pat	127 Kb Duplication, paternal origin containing 2 OMIM genes.
8035	14q32.33(105103181_105339238)x1 mat	236 Kb Deletion, maternal origin, containing 5 OMIM genes.
8989	8q22.2(100072320_100155410)x3 pat	83 Kb Duplication, paternal origin, covering part of the gene *VPS13B*.
9047	11p11.2(47940952_48388755)x3 mat	447 Kb Duplication, maternal origin, containing one OMIM gene.
9059	16p13.2(8861856_8911369)x1 mat	49 Kb Deletion, maternal origin, containing 2 OMIM genes.
9059	Xq11.2(64403462_64454070)x0 pat	50 Kb Nullisomy in the Xq 11.2 region, paternal origin.
8875	17p11.2(19515340_19536015)x1 mat	20 Kb Deletion, maternal origin.
7642	14q11.2(22299149_22968274)x3 pat	669 Kb Duplication, paternal origin, containing 2 OMIM genes.
7642	18q21.1(47765082_47953251)x3 mat	68 Kb Duplication, maternal origin, containing 2 OMIM genes.
8130	5q23.1(118463732_118610549)x3 pat	143 Kb Duplication, paternal origin, containing 2 OMIM genes.
8130	6q16.1(95478783_95554367)x1 mat	75 Kb Deletion, maternal origin.
